# Separation and Enrichment of Antioxidant Peptides from Whey Protein Isolate Hydrolysate by Aqueous Two-Phase Extraction and Aqueous Two-Phase Flotation

**DOI:** 10.3390/foods8010034

**Published:** 2019-01-18

**Authors:** Bin Jiang, Jiaxin Na, Lele Wang, Dongmei Li, Chunhong Liu, Zhibiao Feng

**Affiliations:** Department of Applied Chemistry, Northeast Agricultural University, Harbin 150030, China; jiangbin@neau.edu.cn (B.J.); 18345342585@163.com (J.N.); 18846127496@163.com (L.W.); lidongmei@neau.edu.cn (D.L.); liuchunhong@neau.edu.cn (C.L.)

**Keywords:** ATPE, ATPF, antioxidant peptides, MALDI-TOF MS, RP-HPLC

## Abstract

At present, peptides are separated by molecular exclusion chromatography and liquid chromatography. A separation method is needed in any case, which can be scaled up for industrial scale. In this study, aqueous two-phase extraction (ATPE) and aqueous two-phase flotation (ATPF) were applied to separate and enrich antioxidant peptides from trypsin hydrolysates of whey protein isolates (WPI). The best experimental conditions were investigated, and the results were evaluated using the 2,2′-Azinobis-(3-ethylbenzthiazoline-6-sulphonate) (ABTS) free radical scavenging activity of the peptides-per-unit concentration and the recovery rate (*Y*) of peptides in the top phase of both ATPE and ATPF. Under optimal conditions, the *Y* and ABTS free radical scavenging activity per unit concentration in top phase of ATPE could reach 38.75% and 12.94%, respectively, and in ATPF could reach 11.71% and 29.18%, respectively. The purified peptides were characterized by matrix-assisted laser desorption ionization time-of-flight mass spectrometry (MALDI-TOF MS) and reversed-phase high-performance liquid chromatography (RP-HPLC). PeptideCutter and PeptideMass were applied to analyze and calculate the peptide sequencing. KILDKVGINYWLAHK, VGINYWLAHKALCSEK, and TPEVDDEALEKFDKALK sequences having antioxidant activity were detected in the top phase of ATPE, and VGINYWLAHKALCSEK, KILLDKVGINYWLAHK, ILLDKVGINYWLAHK, IIAEKTKIPAVFK, KIIAEKTKIPAVFK, and VYVEELKPTPEGDLEILLQK sequences having antioxidant activity were detected in the top phase of ATPF. In conclusion, antioxidant peptides were successfully separated from the WPI hydrolysate by ATPE and ATPF; compared with ATPE, ATPF has superior specificity in separating antioxidant peptides.

## 1. Introduction

Whey, the by-product in the manufacturing process of casein, has a large production and high organic content, which is commonly regarded as an environmental problem whose disposal causes difficulty for the dairy industry. Whey protein isolate (WPI) is one of the most plentiful proteins isolated from whey [1]. WPI, a mixture of globular proteins, is mainly composed of α-lactalbumin(α-La) and β-lactoglobulin (β-Lg), at circa 80% [2]. Nowadays, antioxidant peptides obtained from WPI by hydrolysis are widely researched [3]. Studies have shown that antioxidant peptides scavenge free radicals and protect the body from the onslaught of stressors. Antioxidant peptides can protect against damage from reactive oxygen species that alter membrane lipids, deoxyribonucleic acid (DNA) and proteins, and play important roles in cardiovascular diseases, Alzheimer’s, diabetes mellitus, and cancer [4]. Therefore, developing an effective method of isolating antioxidant peptides from WPI can not only produce antioxidant peptides, but also reduce environmental pollution by effectively using food by-products.

According to the characteristic of peptides, some methods are used to fractionate and analyze peptides, such as ultrafiltration, chromatographic methods, mass spectrometry, and electrophoresis. In terms of the separation effect, chromatographic methods are efficient to isolate peptides. Boudesocque et al. used mixed ion-exchange centrifugal partition chromatography to separate antioxidant peptides in the hydrolysate of a complex alfalfa white protein concentrate [5]. Ortiz et al. purified an anti-inflammatory peptide named lunasin by ion-exchange chromatography and size exclusion chromatography [6]. Likewise, peptides were further separated by affinity chromatography. Bah et al. separated the peptides from the hydrolysis products of cattle plasma by gel permeation chromatography [7]. Nevertheless, it is difficult to apply the chromatographic methods mentioned above in industrial production, due to the complex operation process, as well as the time-consuming and expensive instrument cost. Therefore, it is necessary to find appropriate approaches for industrial production.

Aqueous two-phase extraction (ATPE), a liquid–liquid fractionation technique, has been effectively used in the separation, extraction, purification, and enrichment of some biomolecules. Advantages of ATPE include enhanced selectivity, scale-up, process integration, continuous operation, low toxicity and biocompatibility [8]. Jiang et al. reported that the antioxidant peptides from the hydrolysate of WPI were separated using ATPE formed by a propylene oxide polymer (EOPO) co-polymer and phosphate [9]. Hence, purifying bioactive peptides from the mixed peptides by ATPE is achievable. Although ATPE has many advantages, as mentioned above, there are still some problems, such as the large amount of polymers and salts, and its easy to emulsion [10]. With the continuous progress of technology, the aqueous two-phase flotation (ATPF) based on aqueous two-phase system (ATPS) has acquired much attention. Compared with ATPE, ATPF has the advantages of excellent purification efficiency, high concentration coefficients, and low consumption of organic solvents [10]. Thus far, many applications of ATPF were reported, such as the separation of ortho-phenylphenol [11], enicillin G [12], and lincomycin tetracycline [13].

In this study, methods for separating antioxidant peptides were compared, which aim to develop a simple, inexpensive, time-saving, and efficient separation process for the separation of antioxidant peptides from WPI hydrolysates. With this goal, the separation of antioxidant peptides from WPI hydrolysate by 1-propanol/NaH_2_PO_4_ ATPE and ATPF were studied. So far, to our best knowledge, this is the first report to investigate separation of antioxidant peptides by ATPF.

## 2. Materials and Methods

### 2.1. Instruments

High performance liquid chromatograph (Waters E2695, USA) coupled to a C_18_ column and an ultraviolet visible detector (Waters E2695, USA) and matrix-assisted laser desorption ionization time-of-flight mass spectrometry (Bruker Daltonics, USA) were used for characterization. An ultraviolet-visible spectrophotometer (Beijing Purkinje General Instrument Co., Ltd, Beijing, China) was used to determine the concentration of the peptides. An A150011 type vortex mixer (Nanjing Jiajun Biological Co., Ltd., Nanjing, China) and a SC-3610 type low speed centrifuge (Anhui Zhongke Zhongjia Scientific Instrument Co., Ltd., Hefei, China) were applied to promote the formation of the aqueous two-phase extraction. A buoy-type oxygen inhalator (Shandong Huachen high pressure container Co., Ltd., Jinan, China) and a glass rotameter (Shenyang Beixing flow meter factory, Shenyang, China) were used for the preparation of ATPF. A nanoporous ultrafilter with 10 kDa molecular weight cutoffs (Pall Corporation, USA), a CT14D desktop high-speed centrifuge (Shanghai Techcomp Scientific Instrument Co., Ltd., Shanghai, China) and a 300 Da molecular weight cutoff (MWCO) filter (Spectrum Labs, USA) were used for the sample treatment. An FE201EL20 pH meter was utilized to determine the pH of solution, and an AL-04 electronic analytical balance (Mettler Toledo Instruments Co., Ltd., Shanghai, China) was used to weigh.

### 2.2. Reagents

WPI (Shanghai NuoSen Food Trading Co., Ltd.), trypsin 1:10,000 (Beijing Solarbio Science & Technology Co., Ltd.) were used to prepare the WPI hydrolysate. Folin’s phenol reagent (Beijing Solarbio Science & Technology Co., Ltd.), trifluoroacetic acid (TFA), and acetonitrile (Dikma, USA) were applied for the sample analysis. 2,2′-azinobis (3-ethylbenzothiazoline-6-sulphonic acid) (Sigma, USA) and 2,2-diphenyl-1-picrylhydrazyl (Sigma, USA) were utilized to analyze the bioactive peptides. Other reagents covering 1-propanol, NaCl, NaH_2_PO_4_ and so on were analytical reagents.

### 2.3. Preparation of WPI hydrolysate

WPI containing α-La (19%) and β-Lg (73%) determined by High Performance Liquid Chromatography (HPLC) was dissolved in water at a concentration of 5.5% (*w*/*w*). The WPI solution was hydrolyzed with trypsin. The final enzyme-substrate ratio was 1:20 (*w*/*w*). The system was maintained at 37 °C and pH 8 for 24 h [14]. After hydrolysis, the trypsin was deactivated in a boiling water bath for 10 min. The system was cooled to room temperature and centrifuged (4500x g, 30 min) with an ultrafiltration centrifuge tube (10 kDa MWCO) to obtain WPI tryptic peptides [9], which were stored at −20 °C for further analysis.

### 2.4. Separation of Antioxidant Peptides from Wpi Hydrolysate by ATPE and ATPF

ATPEs for separation were prepared by adding the appropriate volume of NaH_2_PO_4_ stock solution, 1-propanol, NaCl, and ultrapure water to obtain an eventual volume of 8 mL in 10 mL graduated centrifuge tubes. After adding WPI hydrolysate (2 mL), ATPEs were mixed fully and centrifuged (700x g, 20 min) to urge phase separation. By determination of the antioxidant activities of peptides in the top and bottom phases, optimum conditions, including the amount of WPI hydrolysate, NaH_2_PO_4_ solution, 1-propanol, and NaCl, were obtained.

**Figure 1:**
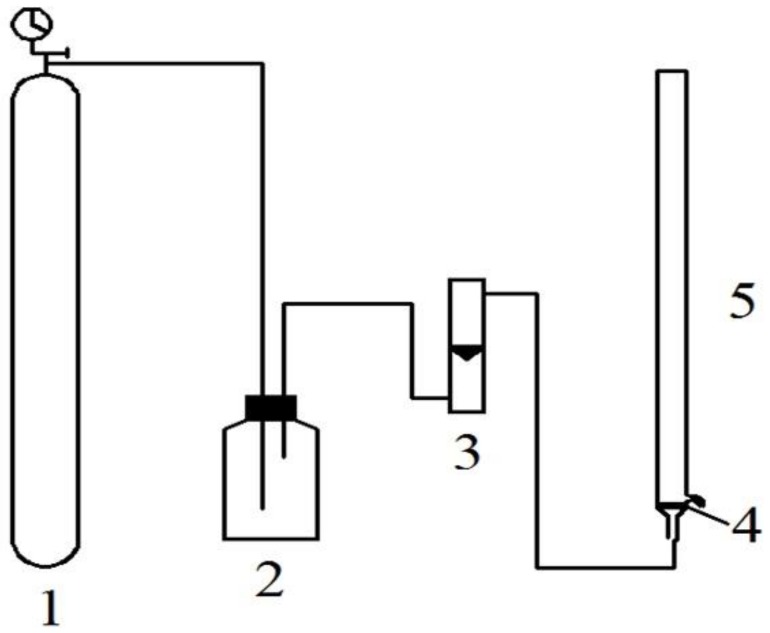
Flotation unit consisted of 1. High-purity nitrogen cylinder; 2. gas buffer device reformed by buoy-type oxygen inhaler; 3. flowrator; 4. G4 glass core; 5. flotation column.

ATPFs were prepared in a flotation column by mixing the appropriate volume of the 20 mg/mL WPI hydrolysate and 15.5% (*w*/*w*) stock solution of NaH_2_PO_4_. When the nitrogen flow rate was stable, an appropriate volume of 1-propanol was added. The flotation unit is described in Figure 1. The gas flow output from the nitrogen cylinder formed a steady flow by gas buffer, which was adjusted by the flowrator. The mixture in the flotation column was separated and enriched by uniform size bubbles. By measuring the antioxidant activities of peptides in the top phases, optimum conditions, including the volume of 1-propanol and WPI hydrolysate, flow rate of nitrogen and flotation time, were obtained.

### 2.5. Determination of the Distribution of Peptides in ATPE and ATPF

The Folin–Ciocalteu phenol reagent was used to determine the concentrations of peptides. The percentage extraction efficiency of peptides in the top phases were calculated using the following Equation (1):

(1) $$Y=\frac{C_{T}V_{T}}{C_{0}V_{0}}\times 100\%$$

where the recovery rate (*Y*) was the recovery rate of peptides in the top phase, while *C*_T_ and *C*_0_ were the concentration of peptides in the top phase and the WPI hydrolysate respectively, *V*_T_ was the top phase volume, and *V*_0_ was the volume of the WPI hydrolysate added to the ATPE or ATPF.

### 2.6. Purification of Peptides

The mixtures from the top and bottom phases of ATPE and ATPF were concentrated by reduced pressure distillation and 1-propanol was removed simultaneously. The concentrate was placed in dialysis bag (300 Da) for 48 h. After dialysis, a lyophilizer was used to get the solid particles called purified peptides. The purified peptides were stored in −20 °C after solid milling for subsequent characterization and determination of antioxidant activity.

### 2.7. Determination of Antioxidant Activity of Purified Peptides

The experimental method based on Venkatesan et al., [15] was applied to the determination of the antioxidant activity of the purified peptides. Ferric reducing antioxidant power,·hydroxyl (OH) radical scavenging activity, ABTS radical scavenging activity, and 2,2-diphenyl-1-picrylhydrazyl (DPPH) radical scavenging activity of the peptides were studied to explore the antioxidant activity of peptides of the WPI hydrolysate and both phases of ATPE and ATPF.

### 2.8. RP-HPLC and MALDI-TOF MS Analysis for the Purified Peptides

Reversed-phase high-performance liquid chromatography (RP-HPLC) coupled to an ultraviolet visible detector was applied to analyze the peptides in each phase and the WPI hydrolysate. The experimental conditions are as follows: Waters C_18_ column (150 mm × 4.6 mm, 5 μm), mobile phase A using 0.1% TFA in acetonitrile, mobile phase B using 0.1% aqueous TFA, detection wavelength from 200 nm to 300 nm, and a flow rate of 1.00 mL/min. Gradient elution process: the concentration of mobile phase A was improved from 2% to 31.5% within 49 min, which was dropped from 31.5% to 2% in 11 min.

The samples were dispersed in ultrapure water (0.2 mg/mL) and mixed 1:1 with a-cyano-4-hydroxycinnamic acid solution (CHCA) dissolved in 70% acetonitrile and 0.1% TFA, which were detected using a Bruker Microflex Linear TOF mass spectrometer instrument with the quality of the range of 500–4000.

## 3. Results and Discussion

### 3.1. Factors on Partitioning of Antioxidant Peptides by ATPE

**Figure 2:**
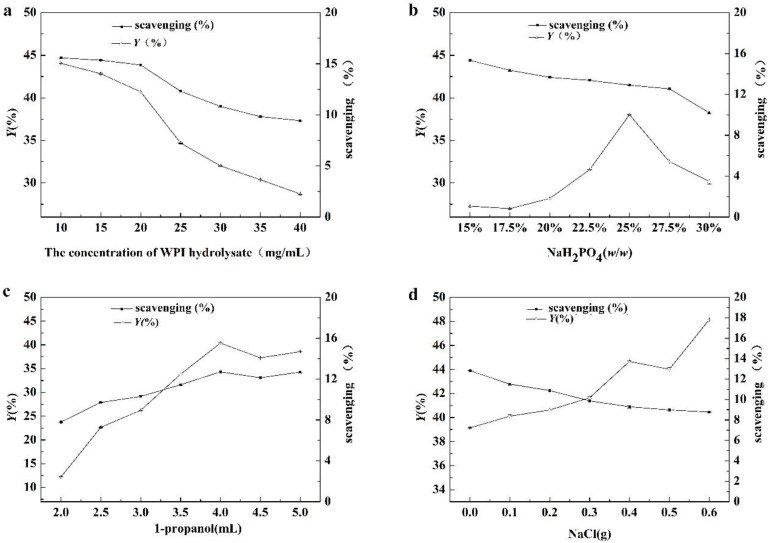
The effects of the concentration of (**a**) whey protein isolate (WPI) hydrolysate, (**b**) the mass fraction of NaH_2_PO_4_, (**c**) 1-propanol and (**d**) NaCl on the separation of antioxidant peptides. The scavenging was 2,2′-Azinobis-(3-ethylbenzthiazoline-6-sulphonate) (ABTS) free radical scavenging activity per unit concentration in top phase of Aqueous two-phase extraction (ATPE).

The effect of WPI hydrolysate concentration, NaH_2_PO_4_ concentration, 1-propanol content, and NaCl were indicated in Figure 2. As indicated in Figure 2a, *Y* and ABTS free radical scavenging activity per unit concentration were decreased with the increase of WPI hydrolysate concentration in the ATPE, which indicated that the recovery rate of peptides and selectivity of antioxidant peptides in top phase were decreased. When the WPI hydrolysate concentration was above 20 mg/mL, protein precipitated at the interphase, resulting in a significant decreasing in *Y*. Considering the utilization of raw materials and extraction efficiencies of antioxidant peptides, WPI concentration of 20 mg/mL was selected as the optimal conditions for the separation of the antioxidant peptides.

Figure 2b showed that *Y* increased with the concentration of NaH_2_PO_4_ until the mass fraction reached 25% (*w*/*w*). The NaH_2_PO_4_ concentration influenced the hydrophobicity of ATPE, meaning that the change in salt concentration broke the balance of the two phases. The peptides were driven from a salt-rich phase to an organic phase due to the salting-out effect [16,17]. At mass fraction above 25% (*w*/*w*), *Y* decreased due to the precipitate discarded at the phases interface. The increase of mass fraction of the NaH_2_PO_4_ solution enhanced the extraction of antioxidant peptides and non-antioxidant peptides into the top phase, so ABTS free radical scavenging activity per unit concentration in the top phase was almost constant (decreased slightly). It indicated that the content of NaH_2_PO_4_ in ATPE couldn’t dramatically influence the proportion of antioxidant peptides in the total peptides of the top phase. Thus, to obtain a high recovery rate of antioxidant peptides, a 25% (*w*/*w*) NaH_2_PO_4_ solution was selected as the optimum condition.

As indicated in Figure 2c, the changing trends of *Y* and ABTS free radical scavenging activity per unit concentration were almost identical. When the content of 1-propanol was present in 4 mL, both *Y* and ABTS free radical scavenging activity per unit concentration reached the highest value, which meant that the recovery rate of antioxidant peptides was at its highest at 4 mL; thus, 4 mL of1-propanol content was chosen as the optimal condition for the extraction of the antioxidant peptides from WPI hydrolysate.

NaCl could change the selectivity of ATPS [18]. As shown in Figure 2d, *Y* increased and ABTS free radical scavenging activity per unit concentration decreased as the amount of NaCl increased. The results showed that more peptides were extracted into the top phase, however, the selectivity of the antioxidant peptides in the top phase of the ATPE was weakened in the presence of NaCl. The finding showed that the addition of NaCl was not a necessary factor to the extraction of the antioxidant peptides from the WPI hydrolysates.

### 3.2. Factors on Partitioning of Antioxidant Peptides by ATPF

**Figure 3:**
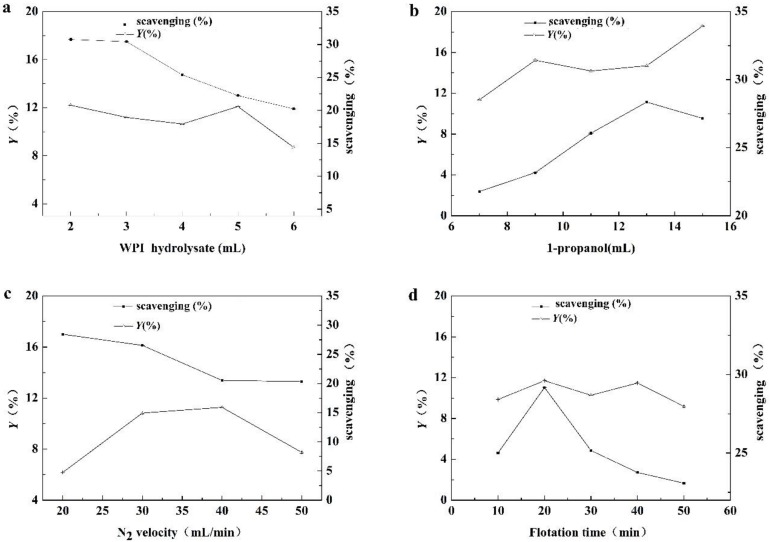
The effects of the volume of (**a**) WPI hydrolysate, (**b**) 1-propanol, (**c**) N_2_ velocity and (**d**) flotation time on the separation of antioxidant peptides. The scavenging was ABTS free radical scavenging activity per unit concentration in top phase of aqueous two-phase flotation (ATPF).

The effect of the added volume of WPI hydrolysate is described in Figure 3a. With the increase of the volume of WPI hydrolysate, *Y* almost remained constant. However, when the added volume of WPI hydrolysate was more than 5 mL, many peptides precipitated at the interphase and *Y* decreased. The ABTS free radical scavenging activity per unit concentration of peptides in the top phase decreased when increasing the volume of the WPI hydrolysate, which means the selectivity of antioxidant peptides in the top phase decreased. The ABTS free radical scavenging activity per unit concentration at 3 mL was similar with that at 2 mL, that is to say, the proportion of antioxidant peptides in the top phase peptides at 2 mL or 3 mL of the WPI hydrolysate were coincident approximately. Based on the above factors, the optimum condition for adding 3 mL of WPI hydrolysate was to ensure that the proportion of antioxidant peptides in the top phase was high and more raw material was added.

The effect of the volume of 1-propanol is described in Figure 3b. Both *Y* and the ABTS free radical scavenging activity of peptides per unit concentration increased significantly when the volume of 1-propanol increased from 7 mL to 13 mL, meaning that the selectivity on the antioxidant peptides was enhanced. With the volume of 1-propanol above 13 mL, more non-antioxidant peptides were distributed to the top phase. Thus, *Y* increased while the ABTS free radical scavenging activity of peptides per unit concentration in the top phase decreased. To obtain a high purity of antioxidant peptides, the volume of 13 mL was selected as the optimum condition.

The effect of the nitrogen flow rate was described in Figure 3c. With the increasing nitrogen flow rate, *Y* showed the trend of increase and then decrease. The ABTS radical scavenging activity of peptides per unit concentration decreased as the nitrogen flow rate increased, implying that the selectivity of antioxidant peptides in the top phase had decreased. When the nitrogen flow rate was above a value, the phase interface of ATPF was unstable, and the material exchange between top and bottom phases was exacerbated, which caused a decrease in target selectivity in the top phase [19,20]. The *Y* at 30 mL/min was about twice that of the *Y* at 20 mL/min; however, the decrease in the ABTS radical scavenging activity of peptides per unit concentration was not significant. Considering *Y* and the purity of the antioxidant peptides, the optimal condition was selected at the N_2_ velocity of 30 mL/min.

The effect of the flotation time is described in Figure 3d. The flotation time is an important factor in the separation of antioxidant peptides in ATPF [21]. *Y* almost remained stable from 10 min to 50 min. The trend of the ABTS free radical scavenging activity of peptides per unit concentration showed that the antioxidant peptides tended to remain in the top phase even at 20 min. This could be explained by the increasing exchange of ineffective materials between the phase interfaces, which had an adverse effect on the extraction of antioxidant peptides after 20 min. Thus, the optimal condition was selected at a flotation time of 20 min.

### 3.3. Determination of Antioxidant Activity of Purified Peptides

Table 1 indicated that the antioxidative activities of peptides in top phase were far greater than peptides in bottom phase from ATPE. Compared with ATPE, peptides from the top phase of ATPF had higher antioxidant activities. Judging from the analysis of the data, both ATPE and ATPF could be used for the separation of antioxidant active peptides, and the selectivity of ATPF was better.

### 3.4. RP-HPLC Analysis of Purified Peptides

**Figure 4:**
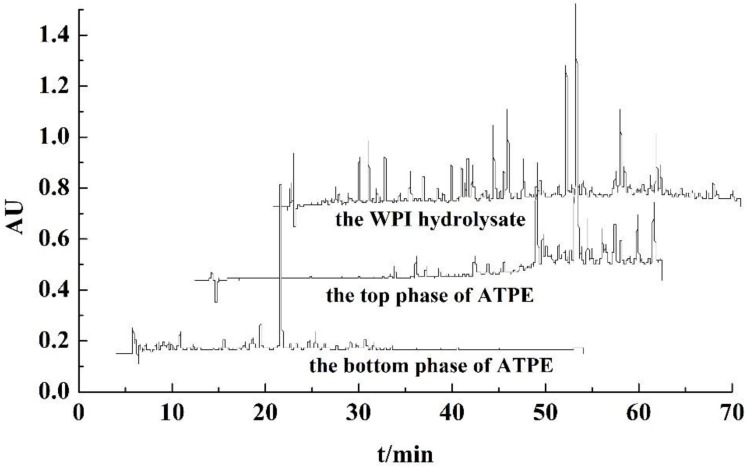
RP-HPLC chromatograms of the WPI hydrolysate, the top and bottom of ATPE under optimal conditions.

According to the chromatograms of purified peptides from ATPE shown in Figure 4, most of the peaks of peptides purified from the top phase were detected with retention times from 36 min to 50 min, and the retention times of remaining peptides ranged from 21 min to 36 min, while most of the peaks of peptides purified from the bottom phase were detected with retention times from 18 min to 30 min. These retention times corresponded to the position of the peptides from the hydrolysate. In the chromatograms of RP-HPLC, the efflux from the C_18_ column comprised the hydrophilic and higher polar peptides had shorter retention times, and the efflux comprised the hydrophobic and lower polar peptides had longer retention times. Peptides purified from the bottom phase in ATPE had shorter retention times, and those from the top phase in ATPE had longer retention times, which proved that more polar and hydrophilic peptides remain in the bottom phase of ATPE, while the less polar and hydrophobic peptides were separated to the top phase of ATPE. Xie et al. discovered that the highly hydrophobic peptides from casein had excellent antioxidant activity [22]. Jiang et al. reported that the highly hydrophobic and less polar peptides had excellent antioxidant activity [9]. Therefore this indicates that peptides from the bottom phase of ATPE had little antioxidant activity and peptides from the top phase of ATPE had excellent antioxidant activity.

**Figure 5:**
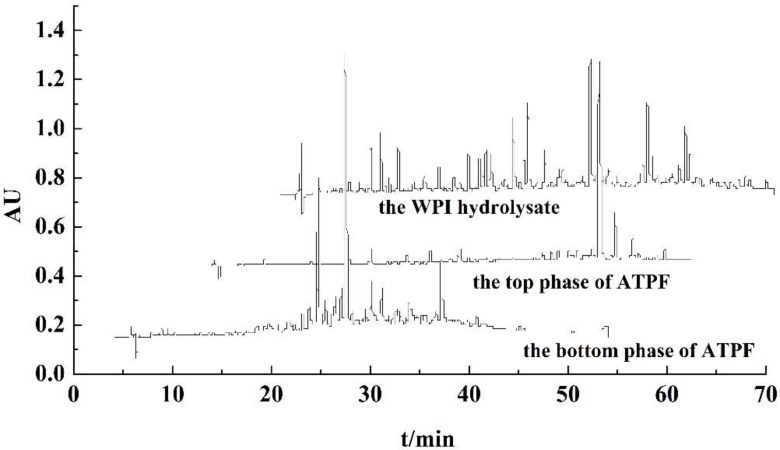
RP-HPLC chromatograms of the WPI hydrolysate, the top and bottom of ATPF under optimal conditions.

According to the chromatograms of purified peptides from ATPF, shown in Figure 5, the retention times of purified peptides from the top phase in ATPF ranged from 35 min to 48 min, and the retention times of purified peptides from the bottom phase were detected before 38 min. Peptides purified from the bottom phase in ATPF had shorter retention times, whereas those from the top phase in ATPF had longer retention times. Thus, the peptides in the top phase of ATPF had antioxidant activity. Compared with the chromatograms of purified peptides from top phase in ATPE, the chromatograms of purified peptides from the top phase in ATPF had fewer peaks, which further proved that ATPF displays a higher selectivity for separation of antioxidant peptides. The flow of bubbles carried hydrophobic peptides into the ATPF phase. It greatly reduced the mixing and mass transfer between the two phases [23]. Therefore, the kinds of peptides in the top phase were simple, whereas the peptides remaining in the bottom phase were more complex.

In comparing the peaks of the WPI hydrolysate peptides, some peaks were not found in the chromatograms of peptides in both phases of ATPE and ATPF, meaning that a few of the peptides were lost during separation and purification. The solubility of some peptides in water decreased after they had been desalted and purified. They were removed by filter with 0.2 μm before they were measured by RP-HPLC. Therefore, the peaks could not be detected by liquid chromatography.

Compared with chromatograms of purified peptides from the WPI hydrolysate, the chromatograms of purified peptides from ATPE and ATPF showed that antioxidant peptides could be extracted and enriched by ATPE and ATPF, and the effect of ATPF was superior than that of ATPE.

### 3.5. MALDI-TOF MS Analysis for Purified Peptides

**Figure 6:**
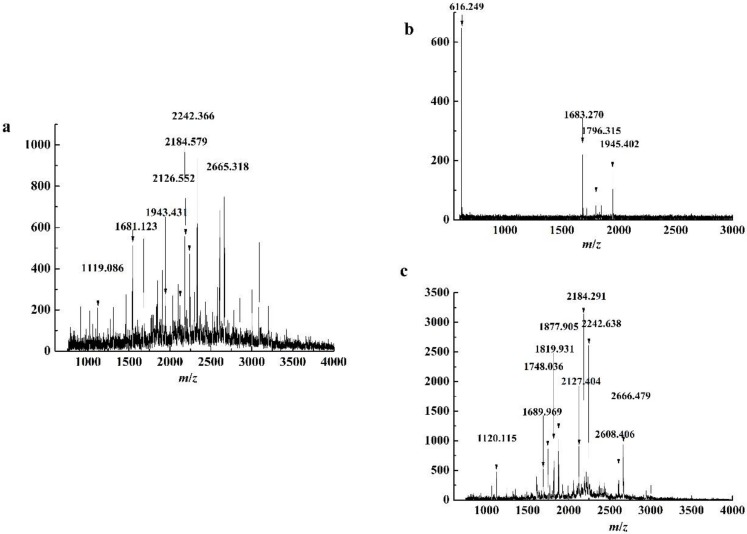
MALDI TOF spectrum of peptides purified of (**a**) WPI hydrolysate, (**b**) top phase and (**c**) bottom phase in ATPE.

PeptideCutter was used to analyze the potential cleavage site of WPI hydrolyzed by trypsin, which was combined with mass spectrometry data to analyze amino acid sequence of peptides [24]. The matrix-assisted laser desorption ionization time-of-flight mass spectrometry (MALDI-TOF MS) spectrum of purified peptides from the WPI hydrolysate and the top and bottom phases of ATPE are presented in Figure 6. WPI is mainly composed of α-La and β-Lg, at circa 80%. The complete amino acid sequences with identified peptide chains of α-La and β-Lg in bovine milk were selected, and the PeptideCutter was used to analyze the potential cleavage sites of WPI by trypsin hydrolysis. The PeptideMass was applied to analyze and calculate the molecular weights of the peptides. The amino acid sequences of the peptides from both phases of the ATPE were identified by compared with the MALDI-TOF MS data [25]. The amino acid sequences of peptides from different source are shown in Table 2. The *m*/*z* was defined as the molecular mass of the peptides measured in mass spectrum.

In addition to α-La and β-Lg, WPI is made up of many other proteins. For this reason, the amino acid sequences of peptides produced by impurities in the WPI hydrolysate couldn’t be identified. Thus, not all amino acid sequences were clarified by MALDI-TOF MS, which caused some MALDI-TOF MS peaks of purified peptides from the top and bottom phases in ATPE not to correspond to those of the WPI hydrolysate. The MALDI-TOF MS peaks of purified peptides from the top phase of ATPE and the bottom phase of ATPE were found in the MALDI-TOF MS peaks of purified peptides from hydrolysate, meaning that the separation and purification process did not change the structure of the peptide sequence significantly.

The KILDKVGINYWLAHK, VGINYWLAHKALCSEK, and TPEVDDEALEKFDKALK sequences, detected in the top phase of ATPE, contain AHK and LK peptides, both of which have an antioxidant activity [26]. However, similar peptides were not found in the bottom phase.

**Figure 7:**
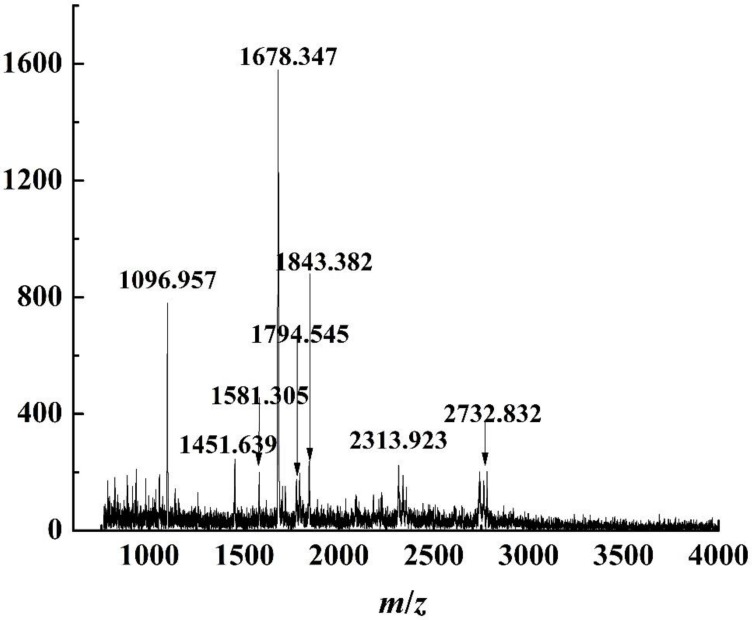
MALDI-TOF spectrum of peptides purified of top phase in ATPF.

The purified peptides from the top phases in ATPF were analyzed using MALDI-TOF MS, as shown in Figure 7. The method of analyzing purified peptides from top and bottom phases in ATPE was used to analyze the purified peptides from ATPF. The peptides sequences are shown in Table 3.

Table 3 showed that the special VGINYWLAHKALCSEK, KILLDKVGINYWLAHK, ILLDKVGINYWLAHK, IIAEKTKIPAVFK, KIIAEKTKIPAVFK, and VYVEELKPTPEGDLEILLQK sequences found in the peptides of the top phase of ATPF contained the AHK, IPAVF [27], YVEEL [9] peptides, all of which have antioxidant activity.

In conclusion, the kinds of antioxidant peptides in the top phase of ATPF had more than that in the top phase of ATPE. It also proved that ATPF had higher selectivity of antioxidant peptides.

### 3.6. Comparison of ATPE, ATPF and Other Methods of Extraction

At present, chromatography is the main method of purification bioactive peptides, including ion-exchange chromatography, affinity chromatography, size exclusion chromatography, hydrophilic interaction liquid chromatography, and ultra-high-pressure liquid chromatography [28]. The main method of purification antioxidant peptides was ultrafiltration combined with consecutive chromatography (size exclusion chromatography, ion-exchange chromatography and reverse-phase chromatography) [4,29]. The antioxidant activity (ABTS radical scavenging activity, DPPH radical scavenging activity, and OH radical scavenging activity) of purified peptides was between 40% and 86%, approximately [30,31,32,33]. However, methods based on chromatography are difficult to apply in industrial production, because the techniques are operationally cumbersome, time consuming, and inherently expensive [28]. Ultrafiltration is difficultly used in a widely, due to ultrafiltration membranes lack selectivity and easily contaminated during ultrafiltration. The details are displayed in Table 4. The purification peptides by ATPE and ATPF are fast, simple, inexpensive, low in toxicity, and biocompatible. In addition to the advantages mentioned above, ATPF also shows enhanced selectivity, scale-up, process integration, continuous operation, and high throughput in separation of complex mixtures. Overall, ATPE and ATPF can maintain the biological activity of antioxidant peptides, with the advantages of convenience, speed, low cost and low pollution, which is more suitable for application in industry.

## 4. Conclusions

In this study, antioxidant peptides were separated by ATPE and ATPF from trypsin hydrolysates of WPI. The optimal conditions for the separation using 1-propanol-NaH_2_PO_4_ ATPE and ATPF were determined by experiment. According to an RP-HPLC analysis of the peptides, both ATPE and ATPF separated antioxidant peptides from the WPI hydrolysate based on the hydrophobicity or polarity of the peptides. Based on MALDI-TOF MS analysis of the purified peptides, the top phase of the ATPE and ATPF were mainly composed of peptides that contained amino acid sequences with antioxidant activity. Compared with other methods of extraction peptides, ATPE and ATPF are fast, simple, inexpensive, have low toxicity, and are biocompatible. Compared with ATPE, ATPF has a better specificity in separating antioxidant peptides. This method can also be scaled up for use on an industrial scale. In summary, ATPF was suitable for the extraction antioxidant peptides from trypsin hydrolysate of WPI. The goal of extraction antioxidant peptides from WPI was achieved to a degree; however, the purification method needs to be further studied.

## Figures and Tables

**Table 1:** Four antioxidant activity levels of peptides after purification.

The Source of Peptides	ABTS Radical Scavenging Activity (%)	DPPH Radical Scavenging Activity (%)	OH Radical Scavenging Activity (%)	Ferric Reducing Antioxidant Power
WPI hydrolysate	53.44 ± 0.53 ^b^	8.34 ± 0.09 ^b^	79.91 ± 0.85 ^b^	0.297 ± 0.001 ^b^
Top phase of ATPE	68.53 ± 0.50 ^c^	15.98 ± 0.03 ^c^	85.98 ± 0.38 ^c^	0.361 ± 0.001 ^c^
Bottom phase of ATPE	34.81 ± 0.49 ^a^	5.98 ± 0.17 ^a^	51.26 ± 0.33 ^a^	0.167 ± 0.003 ^a^
Top phase of ATPF	81.88 ± 0.68 ^d^	23.68 ± 0.07 ^d^	89.72 ± 0.64 ^d^	0.403 ± 0.001 ^d^

The data (mean ± standard deviation) derived from three replicates, as described in the Materials and Methods section. **a**–**d** means in the same column with different superscripts are significantly different (*P* ˂ 0.01); ABTS: 2,2′-Azinobis-(3-ethylbenzthiazoline-6-sulphonate); DPPH: 2,2-diphenyl-1-picrylhydrazyl; OH radical: hydroxyl radical (OH radical); WPI: whey protein isolate; ATPE: Aqueous two-phase extraction; ATPF: Aqueous two-phase flotation.

**Table 2:** Amino acid sequence of peptides of different phases in ATPE.

Peptide Source	Amino Acid Sequence	Protein Fragment	*m*/*z*	Antioxidant-Active Peptides
Top phase of ATPE	KILDK	α-La (113–117)	616.249	
Bottom phase of ATPE	LDQWLCEKL	α-La (115–123)	1120.115	
Top phase of ATPE	ALCSEKLDQWLCEK	α-La (109–122)	1683.270	
Bottom phase of ATPE	LSFNPTQLEEQCHI	β-Lg (149–162)	1689.969	
Bottom phase of ATPE	WENGECAQKKIIAEK	β-Lg (61–75)	1748.036	
Top phase of ATPE	KILDKVGINYWLAHK	α-La (94–108)	1796.315	AHK
Top phase of ATPE	VGINYWLAHKALCSEK	α-La (99–114)	1846.401	AHK
Bottom phase of ATPE	NDQDPHSSNICNISCDK	α-La (63–79)	1877.905	
Top phase of ATPE	TPEVDDEALEKFDKALK	β-Lg (125–141)	1945.402	LK
Bottom phase of ATPE	FLDDDLTDDIMCVKKILLDK	α-La (80–98)	2242.638	
Bottom phase of ATPE	YLLFCMENSAEPEQSLACQCLVR	β-Lg (102–124)	2666.749	

**Table 3:** Amino acid sequence of peptides of top phase in ATPF.

Peptide Source	Amino Acid Sequences	Protein Fragment	*m*/*z*	Antioxidant-Active Peptide
Top phase of ATPF	IIAEKTKIPAVFK	β-Lg (71–83)	1451.639	IPAVF
Top phase of ATPF	KIIAEKTKIPAVFK	β-Lg (70–83)	1581.305	IPAVF
Top phase of ATPF	ILLDKVGINYWLAHK	α-La (95–108)	1678.347	AHK
Top phase of ATPF	KILLDKVGINYWLAHK	α-La (94–108)	1794.545	AHK
Top phase of ATPF	VGINYWLAHKALCSEK	α-La (99–114)	1843.382	AHK
Top phase of ATPF	VYVEELKPTPEGDLEILLQK	β-Lg (41–60)	2313.923	YVEEL
Top phase of ATPF	TPEVDDEALEKFDKALPMHIR	β-Lg (125–148)	2732.832	

**Table 4:** Comparison of ATPE, ATPF and other methods of extraction.

Method of Extraction	Advantage	Limitation
Ion-exchange chromatography [6]	The separation of highly cationic or anionic peptides.	Requires complementary steps for the separation and low selectivity.
Affinity chromatography [34]	The separation of different types of peptides.	physicochemical properties of the ligands yet to be discovered.
Size exclusion chromatography [35]	Mild elution conditions, with minimal impact on the conformational structure.	High column requires separation of mixed peptides.
Hydrophilic interaction liquidChromatography [36]	The method shows great potential for the separation of short peptide sequences (5 amino acids).	Limited flexibility and applicability, poorly understood problems with sample solubility and the retention mechanisms.
Ultra-high-pressure liquid chromatography [37]	Increased throughput, resolution, and sensitivity in separation of complex protein mixtures.	Ultra-high pressures increase chromatographic band broadening and compromise efficiency of the column.
Ultrafiltration	Short time, high throughput, and high recovery.	Difficult to control experimental conditions in the membrane.
ATPE [9,38]	Rapid, simple, and inexpensive, low in toxicity and biocompatibility separation process.	Large amounts of polymers and salts and easy to emulsify.
ATPF	Increased throughput in separation of complex protein mixtures. Enhanced selectivity, scale-up, process integration, continuous operation, low toxicity, and biocompatibility.	Large amounts of polymers and salts
